# Stereotactic body radiotherapy: a new non-invasive way to conduct pulmonary artery denervation

**DOI:** 10.3389/fmed.2025.1607638

**Published:** 2025-06-25

**Authors:** Manzhen Liao, Taoyue Yao, Yonghui Xie, Shelby Kutty, Jinqiao Liu, Wei Peng, Ting Huang, Huaiyang Chen, Haoqin Fan, Zhenghui Xiao, Qiming Liu, Yunbin Xiao

**Affiliations:** ^1^Academy of Pediatrics, University of South China, Changsha, Hunan, China; ^2^Department of Cardiology, The Affiliated Children’s Hospital of Xiangya School of Medicine, Central South University (Hunan Children’s Hospital), Changsha, Hunan, China; ^3^Department of Ultrasound, The Affiliated Children’s Hospital of Xiangya School of Medicine, Central South University (Hunan Children’s Hospital), Changsha, Hunan, China; ^4^Department of Ultrasound, The Affiliated Nanhua Hospital, University of South China, Hengyang, Hunan, China; ^5^Pediatric and Congenital Cardiology, Taussig Heart Center, Johns Hopkins School of Medicine, Baltimore, MD, United States; ^6^Department of Intensive Care Unit, The Affiliated Children’s Hospital of Xiangya School of Medicine, Central South University (Hunan Children’s Hospital), Changsha, Hunan, China; ^7^Department of Cardiovascular Medicine, Second Xiangya Hospital, Central South University, Changsha, China

**Keywords:** pulmonary arterial hypertension, stereotactic body radiotherapy, pulmonary artery denervation, sympathetic nerves, acute thromboembolic pulmonary arterial hypertension

## Abstract

**Background:**

Pulmonary arterial hypertension is a severe and life-threatening disease characterized by a progressive increase in pulmonary vascular resistance. Catheter-based pulmonary artery denervation (PADN) has been conducted in pulmonary arterial hypertension patients; however, if stereotactic body radiotherapy (SBRT) can become a new non-invasive way to conduct, PADN has not been elucidated.

**Methods:**

A total of 12 healthy male New Zealand rabbits were digitally marked on their foreheads and randomly divided into the control group (*n* = 6) and SBRT group (*n* = 6) at a ratio of 1:1 using computer-generated random numbers. In the SBRT group, rabbits were treated with a single dose of 15 Grey and then bred for a minimum of 3 months. The rabbit models of acute thromboembolic pulmonary arterial hypertension (ATEPAH) were established by injecting autologous blood clots into the femoral vein. Right ventricular function and hemodynamics were assessed by echocardiography and right heart catheterization. Pulmonary artery sympathetic nerves were evaluated by pathological staining.

**Results:**

The SBRT procedure was successfully performed in all six rabbits. Compared to the control group, SBRT-PADN reduced pulmonary artery systolic pressure and mean pulmonary artery pressure in ATEPAH rabbits. Meanwhile, SBRT-PADN could attenuate pulmonary artery dilatation in ATEPAH rabbits. Histological examination revealed evident structural damages in sympathetic nerves of SBRT-PADN animals, including vacuolization, nuclear pyknosis, and digestion chambers. No adverse events had been observed, and sparing of pulmonary artery of the intima and media was detected up to 90 days post-procedure.

**Conclusion:**

SBRT could destroy sympathetic nerves around pulmonary artery locally, which may represent a novel option for performing PADN. In addition, this study provided its short-term effectiveness and safety.

## Graphical abstract

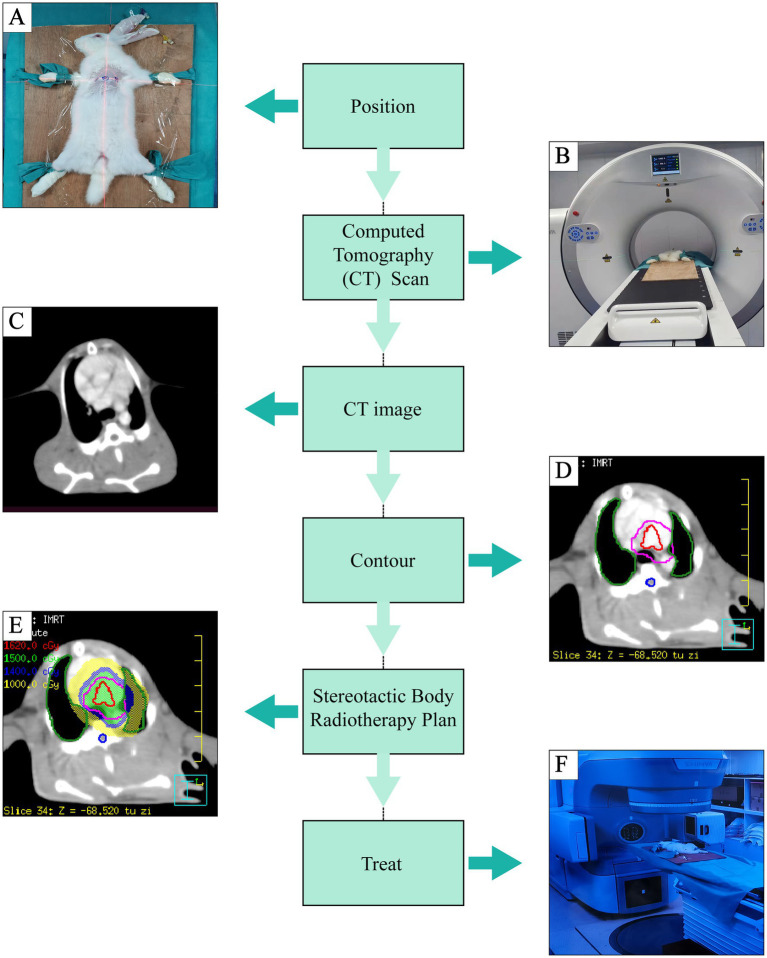

## Introduction

Pulmonary arterial hypertension (PAH) is a severe and life-threatening disease characterized by a progressive increase in pulmonary vascular resistance, ultimately resulting in right ventricular failure and death (1–3). Long-term use of targeted medications and their associated complications impose a significant societal and economic burden. Moreover, current targeted pharmacotherapies have yielded unsatisfactory outcomes (3, 4). Numerous studies have demonstrated that sympathetic nervous system (SNS) overactivity plays a crucial role in the occurrence and development of PAH (5–9). Pulmonary artery denervation (PADN) is a new catheter-based ablation technique that aims to suppress the overactive SNS. This technique has shown promise in decreasing mean pulmonary artery pressure (mPAP) and pulmonary vascular resistance (PVR) (4, 10–14). Although the majority of existing studies have focused on ultrasound and radiofrequency ablation and have shown support for the clinical and cost-effectiveness of this therapy, the transluminal approaches have several limitations. These limitations include risk of local endothelial damage, inaccuracy in locating targets, time-consuming procedures, and invasive nature of the technique (11–17). Hence, we have gradually commenced exploring alternative non-invasive technology that are suitable for PADN.

Our previous study had proved high-intensity focused ultrasound (HIFU) could non-invasively destroy SNs around PA in rabbit models effectively and safely, while the accuracy of HIFU with PADN need improved (18). Stereotactic body radiotherapy (SBRT) demonstrated exceptional accuracy and precision, delivering non-invasive and high-dose radiation therapy to the target area, while minimizing injury to surrounding normal tissues (19). SBRT was usually used for treatment of solid tumors. In recent years, advances in radiation technology have expanded the application of SBRT beyond oncology, including non-oncological fields such as ventricular tachycardia (20), hypertension (19), and hypertrophic cardiomyopathy (21, 22). Cai et al. have demonstrated the efficacy of SBRT in injuring sympathetic nerves (SNs) around renal artery, thereby treating hypertension without causing harm to surrounding tissues (19). Furthermore, Zhu et al. have shown that SBRT irradiation of porcine ventricular septum was safe and feasible, providing valuable insights for clinical application (21). In March 2021, this technology was first employed in human treatment research (22). To date, five cases have been studied, demonstrating alleviation of clinical symptoms in patients with hypertrophic obstructive cardiomyopathy during 1 year of follow-up. No radiation-related complications or adverse events have been reported (21). A recent study demonstrates that SBRT can achieve complete, circumferential PADN in an acute canine model of PAH (23).

However, there are few reports addressing the potential of SBRT as a novel denervation method for the treatment of PAH. Therefore, this study aimed to investigate the feasibility of SBRT for conducting PADN.

## Materials and methods

### Animals

New Zealand White rabbits (*n* = 12, male, 2.0–2.5 kg) were provided by Hunan Taiping Biology Science and Technology Co., Ltd., China [License number: SCXK (Xiang) 2020–0005]. All rabbit experiments were approved by the Laboratory Animal Ethics Committee at the Hunan University of Chinese Medicine (Number: 2022030101) and in accordance with the guidelines from Directive 2010/63/EU of the European Parliament on the protection of animals used for scientific purposes. All rabbits were housed at 10–22°C in humidity-controlled room with a 12:12-h light–dark cycle and provided unrestricted water and a normal chow diet. After a 7-day acclimatization to laboratory conditions, all rabbits were digitally marked on their foreheads and randomly divided into the control group (*n* = 6) and the SBRT group (*n* = 6) at a ratio of 1:1 using computer-generated random numbers. The procedures were performed under 2% pentobarbital sodium (30 mg/kg, Sigma) anesthesia via marginal ear vein. All rabbits had spontaneous breathing by a mask to absorb oxygen. In addition, a rabbit model of acute thromboembolic pulmonary arterial hypertension (ATEPAH) was established by injecting autologous blood clots into the femoral vein (FV) using a 5F catheter. Animals were euthanized by marginal ear vein injection of an overdose of sodium pentobarbital (100 mg/kg, Sigma) prior to the removal of tissues for analysis. Detailed experimental designs are depicted in Supplementary Figure 1.

### SBRT procedure for PADN

All rabbits were fixed in supine position and underwent positioning scan by large aperture computed tomography (CT) simulator (Shinva Medical Instrument, Shandong, China). The CT reference points were marked on the skin with three metallic radiopaque markers at the level of PA using wall-mounted lasers. Rabbit’s body was hold by sticky tapes to minimize movement during anesthesia. Contrast medium (iomeprol 350 mg I/ml, 2 mL/kg) was injected through a cannulated ear vein to enhance scans and identify the PA (20-s delay, flow rate 0.2 mL/s). After PA enhanced CT, all images were imported to a data management workstation to outline the circumferential areas around the left and right pulmonary arteries to determine clinical target volume (CTV). To account for setup and radiation delivery uncertainties, an isotropic 2-mm expansion on CTV was used to determine the planning target volume (PTV). Adjacent organs at risk (OARs), such as spinal cord and lungs, were delineated to protect them from radiation. SBRT radiation treatment plan was generated in the Philips pinnacle treatment planning system (TPS) to deliver a single fraction irradiation of 15 Grey (Gy) to the PTV. The treatment was delivered using an image-guided radiotherapy-equipped XHA1400 system (Shinva Medical Instrument, Shandong, China). Rabbits in the SBRT group underwent treatment and were bred for a minimum of 3 months (19, 21, 22, 24, 25). The procedure workflow was shown in the Central Illustration.

### Echocardiography

All rabbits underwent transthoracic echocardiography (TTE) performed by an experienced sonographer using a EPIQ 7C Color Doppler Ultrasonography (Philips Medical Systems, Netherlands) with a X12-8 transducer, frequency ranging from 8 to 12 MHz. Pulmonary artery diameter (PAD) was measured in parasternal short-axis view (PSAX) and then using a pulsed-wave (PW) Doppler to measure pulmonary artery acceleration time (PAAT). Four chamber dimensions were conducted from apical four chamber (A4C) and then measuring tricuspid annular plane systolic excursion (TAPSE) by M-mode ultrasound in same view. The electrocardiogram (ECG) was synchronously recorded. Each rabbit’s measurements were averaged from three consecutive cardiac cycles. All measurements were carried out according to the guidelines for the American Society of Echocardiography (26).

### Hemodynamic measurements or right heart catheterization

First, a 5F catheter was inserted into right FV to collect 2 mL of venous blood. The blood sample was then mixed with 50 U thrombin and left to stand for 2 h to prepare the blood clot that was used for pulmonary embolization. As a result, a blood clot was formed, which was cut into 2-mm segments and suspended in 5 mL of saline solution (27). Subsequently, after right external jugular vein isolated, a 5F right heart catheter filled with heparinized saline (10 U/ mL) was inserted into it. The catheter was connected to BL-420F Biofunctional Experiment System (Chengdu Techman Co., Ltd., China) via a pressure sensor monitoring pressure changes. Guided by ultrasonic images, the catheter was advanced through superior vena cava, right atrium (RA), right ventricle (RV), and finally into PA.

Hemodynamic parameters, such as pulmonary artery systolic pressure (PASP), pulmonary artery diastolic pressure (PADP), and mPAP, were measured. In addition, the pressure difference between pre- and post-embolization (pressure difference = post-embolism pressure value—pre-embolism pressure value) was calculated and compared. If the waiting time for pressure measurement operation exceeded a certain threshold, heparinized saline was injected every 15 min to flush the tube. Subsequently, thrombus suspension was gradually injected into FV using a 5F catheter. BL-420F simultaneously recorded PASP until a reading of 30 mmHg, which was the criterion of PAH.

### Histopathological assessment

Gross examination of the pulmonary arteries and surrounding tissues was conducted. A tissue block consisting of the main pulmonary artery (MPA), pulmonary artery bifurcation, and surrounding connective tissue was dissected and immersed in 4% paraformaldehyde for at least 24 h. Samples were embedded in paraffin, cut to a thickness of 4 μm, and stained with hematoxylin and eosin (HE). Samples containing lung vessels were additionally stained with Modified Russell-Movat (Movat) pentachrome. Ablation lesions in peri-pulmonary arterial nerves could be evaluated using a semi-quantitative ordinal grading system. This system enabled the assessment of nerve lesion severity on a five-point scale: 0 (no lesions), 1 (minimal lesions), 2 (mild lesions), 3 (moderate lesions), and 4 (severe lesions). In addition, the system effectively evaluated endoneurial changes, such as vacuolation, pyknotic nuclei, and digestion chambers (19, 24, 28, 29).

### Immunohistochemical assay

Tyrosine hydroxylase acts as a marker for identifying sympathetic nerve fascicles (30, 31). After the histological evaluation of all slides, sections from pulmonary arteries in each group were chosen for subsequent immunohistochemical staining against tyrosine hydroxylase (TH) (dilution 1:100, NB300-110SS, Novus). The average optical density values (integrated optical density/area) of SNs were measured by Image Pro-plus 6.0 software.

### Statistical analysis

All analyses were analyzed by SPSS 27.0. Measurement data were presented as mean ± standard deviation (SD). The independent samples *t*-test was used to analyze data with normal distribution and homogeneous variances between two groups. In addition, the paired-sample *t*-test was employed to compare differences between same group before and after modeling. Non-normally distributed quantitative variables were compared using the Mann–Whitney *U* test. A *p*-value of < 0.05 was indicative of statistical significance.

## Results

### Procedural outcomes

In this study, SBRT was performed in a group of rabbits, and all 12 rabbits successfully completed the anticipated-in-life phase, resulting in 100% survival. The treatment characteristics are summarized in Supplementary Table 1. Supplementary Figure 2 illustrated a typical PADN plan, demonstrating the precise delivery of a 15 Gy dose to targeted regions surrounding the pulmonary arteries with a rapid dose falloff to adjacent tissues. The collective ablation target volume was determined to be 2.4 ± 0.5 mL, and the average on-table treatment duration was 7.0 ± 0.9 min.

### Effects of PADN on hemodynamics and RV function in ATEPAH

**Figure 1:**
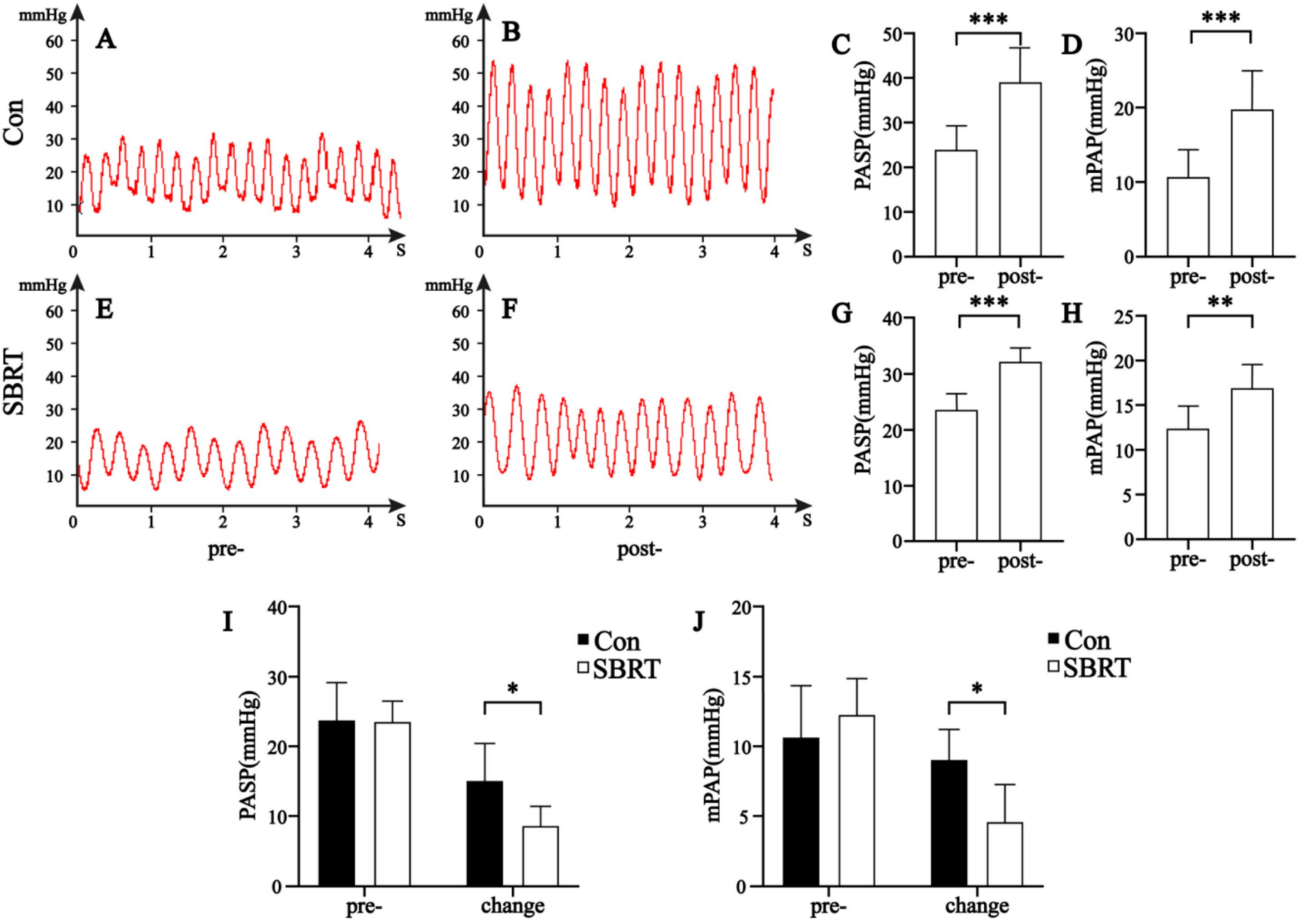
Hemodynamic parameters. Hemodynamic parameters in the control group **(A–D)** and SBRT group **(E–H)** before and after establishing ATEPAH models. Comparisons of PASP **(I)** and mPAP **(J)** between the control group and SBRT group before and after establishing ATEPAH models. Con, control group; SBRT, stereotactic body radiotherapy group; PASP, pulmonary artery systolic pressure; mPAP, mean pulmonary artery pressure. The independent samples *t*-test was used to analyze data between two groups. In addition, the paired-sample *t*-test was employed to compare differences between the same group before and after modeling. Comparison in the same group before and after establishing ATEPAH rabbits: **p < 0.01; ***p < 0.001. Comparison between the control group and SBRT group: *p < 0.05. *n* = 6 rabbits per group for all analyses.

The pre-ATEPAH values of PASP, PADP, and mPAP had no significant differences between the control group and SBRT group (*p* > 0.05; Table 1). Compared with pre-ATEPAH PASP, both two groups showed increased post-ATEPAH PASP which was higher than 30 mmHg (*p* < 0.001; Table 1; Supplementary Figure 3) after injecting thrombus suspension, indicating the establishment of PAH rabbit models was successful. The post-ATEPAH PASP (*p* < 0.001; Table 1), PADP (*p* < 0.01; Table 1), and mPAP (*p* < 0.001; Table 1) in the control group, as well as the post-ATEPAH PASP (*p* < 0.001; Table 1) and mPAP (*p* < 0.01; Table 1) in the SBRT group, increased. No significant difference of PADP was observed in the SBRT group (*p* > 0.05). Furthermore, the elevations of post-ATEPAH PASP and post-ATEPAH mPAP in the SBRT group were significantly lower than that in the control group (*p* < 0.05; Table 1; Figure 1).

**Table 1:** Hemodynamic parameters of ATEPAH rabbits (x_±s, mmHg).

Parameters	Con (*n* = 6)			SBRT (*n* = 6)				
	Preoperative data	Postoperative data	*p*	Preoperative data	Postoperative data	*p*	*P* ^a^	*P* ^b^
PASP	23.72 ± 5.40	38.93 ± 7.75	0.0008	23.57 ± 2.94	32.28 ± 2.43	0.0006	0.9510	0.0222
PADP	0.95 ± 2.90	3.87 ± 2.25	0.0040	2.66 ± 2.82	4.57 ± 2.69	0.1279	0.3243	0.4173
mPAP	10.69 ± 3.65	19.72 ± 5.22	0.0002	12.25 ± 2.61	16.87 ± 2.66	0.0081	0.4155	0.0105

ATEPAH, acute thromboembolic pulmonary arterial hypertension; Con, control group; SBRT, stereotactic body radiotherapy group; PASP, pulmonary artery systolic pressure; PADP, pulmonary artery diastolic pressure; mPAP, mean pulmonary artery pressure. The independent samples t-test was used to analyze data between two groups. In addition, the paired-sample *t*-test was employed to compare differences between the same group before and after modeling. P: comparison in the same group before and after establishing ATEPAH rabbits. P^a^: comparison between the control group and SBRT group before establishing ATEPAH rabbits. P^b^: comparison of pre- and post-establishing differences between the control group and SBRT group.

**Figure 2:**
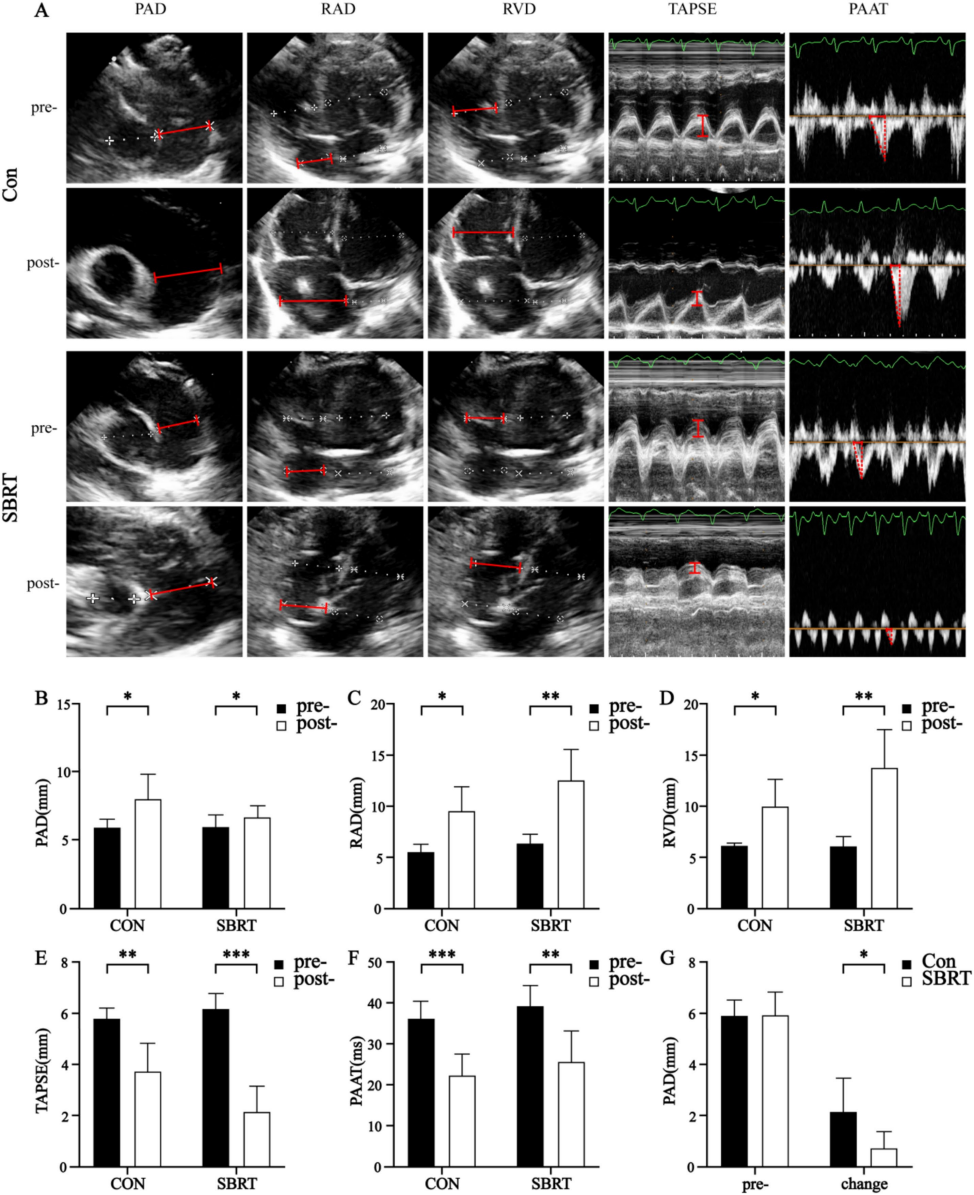
Ultrasonography parameters. Ultrasonography parameters **(A)** in the control group and SBRT group before and after establishing ATEPAH models. Changes of PAD **(B)**, RAD **(C)**, RVD **(D)**, TAPSE **(E)**, and PAAT **(F)** between the control group and SBRT group before and after establishing ATEPAH models. PAD, pulmonary artery diameter; PAAT, pulmonary artery acceleration time; RAD, right atrium diameter; RVD, right ventricle diameter; TAPSE, tricuspid annular plane systolic excursion; Con, control group; SBRT, stereotactic body radiotherapy group. The independent samples *t*-test was used to analyze data between two groups. In addition, the paired-sample *t*-test was employed to compare differences between the same group before and after modeling. Comparisons in same group before and after establishing ATEPAH rabbits: *p < 0.05; **p < 0.01; ***p < 0.001. *n* = 6 rabbits per group for all analyses.

All rabbits completed TTE successfully. There were no significant differences in right atrium diameter (RAD), right ventricle diameter (RVD), left ventricle diameter (LVD), PAD, TAPSE, and PAAT between two groups before injecting autologous blood clots (*p* > 0.05; Table 2). After injecting autologous blood clots, the PA dilated in both groups (*p* < 0.05), while the dilation of PA in the SBRT group was lower than that in the control group (*p* < 0.05; Figure 2). With the rapid increase in PASP, interventricular septal (IVS) flattening occurred. All RVD and RAD enlarged in the control group (*p* < 0.05; Table 2) and SBRT group (*p* < 0.01; Table 2), while there were no obvious changes of left atrium diameter (LAD) in two groups and LVD in the control group (*p* > 0.05; Table 2). Changes of RVD and RAD in end-systolic and end-diastolic were proportional to PASP until acute right heart failure occurred (Figure 2). In addition, TAPSE (*p* < 0.01; Table 2) and PAAT (*p* < 0.001; Table 2) in the control group, as well as TAPSE (*p* < 0.001; Table 2) and PAAT (*p* < 0.01; Table 2) in the SBRT group, decreased. These findings indicate that pulmonary hypertension was associated with right atrial and right ventricular dilation, as well as right ventricular dysfunction. In the SBRT group, there were no significant improvements observed in RAD, RVD, TAPSE, and PAAT. In fact, TAPSE showed a more pronounced decrease. Taken together, these results demonstrated that SBRT improved hemodynamics and attenuated PA dilatation in ATEPAH rabbits.

**Table 2:** Ultrasonography parameters of ATEPAH rabbits (x_±s).

Parameters	Con (*n* = 6)			SBRT (*n* = 6)				
	Preoperative data	Postoperative data	*p*	Preoperative data	Postoperative data	*p*	*P* ^a^	*P* ^b^
RAD, mm	5.52 ± 0.71	9.54 ± 2.37	0.0129	6.32 ± 0.94	12.55 ± 2.97	0.0039	0.1308	0.2083
RVD, mm	6.16 ± 0.30	9.99 ± 2.68	0.0178	6.12 ± 0.95	13.80 ± 3.70	0.0043	0.9151	0.0667
LAD, mm	6.50 ± 0.50	6.46 ± 0.73	0.9120	7.37 ± 0.69	6.02 ± 0.69	0.0551	0.0329	0.0786
LVD, mm	7.99 ± 0.45	9.68 ± 2.64	0.1307	7.67 ± 0.71	6.95 ± 0.52	0.0127	0.3608	0.0163
PAD, mm	5.90 ± 0.61	8.05 ± 1.78	0.0104	5.93 ± 0.90	6.67 ± 0.83	0.0385	0.9416	0.0491
PAAT, ms	36.17 ± 4.22	22.33 ± 5.24	0.0001	39.00 ± 5.29	25.50 ± 7.64	0.0091	0.3291	0.9251
TAPSE, mm	5.78 ± 0.42	3.73 ± 1.10	0.0034	6.17 ± 0.60	2.15 ± 1.00	0.0004	0.2430	0.0110

ATEPAH, acute thromboembolic pulmonary arterial hypertension; Con, control group; SBRT, stereotactic body radiotherapy group; PASP, pulmonary artery systolic pressure; PADP, pulmonary artery diastolic pressure; mPAP, mean pulmonary artery pressure. P: comparison in the same group before and after establishing ATEPAH rabbits. The independent samples *t*-test was used to analyze data between two groups. In addition, the paired-sample *t*-test was employed to compare differences between the same group before and after modeling. P^a^: comparison between the control group and SBRT group before establishing ATEPAH rabbits. P^b^: comparison of pre- and post-establishing differences between the control group and SBRT group.

### Gross and histological evaluation

**Figure 3:**
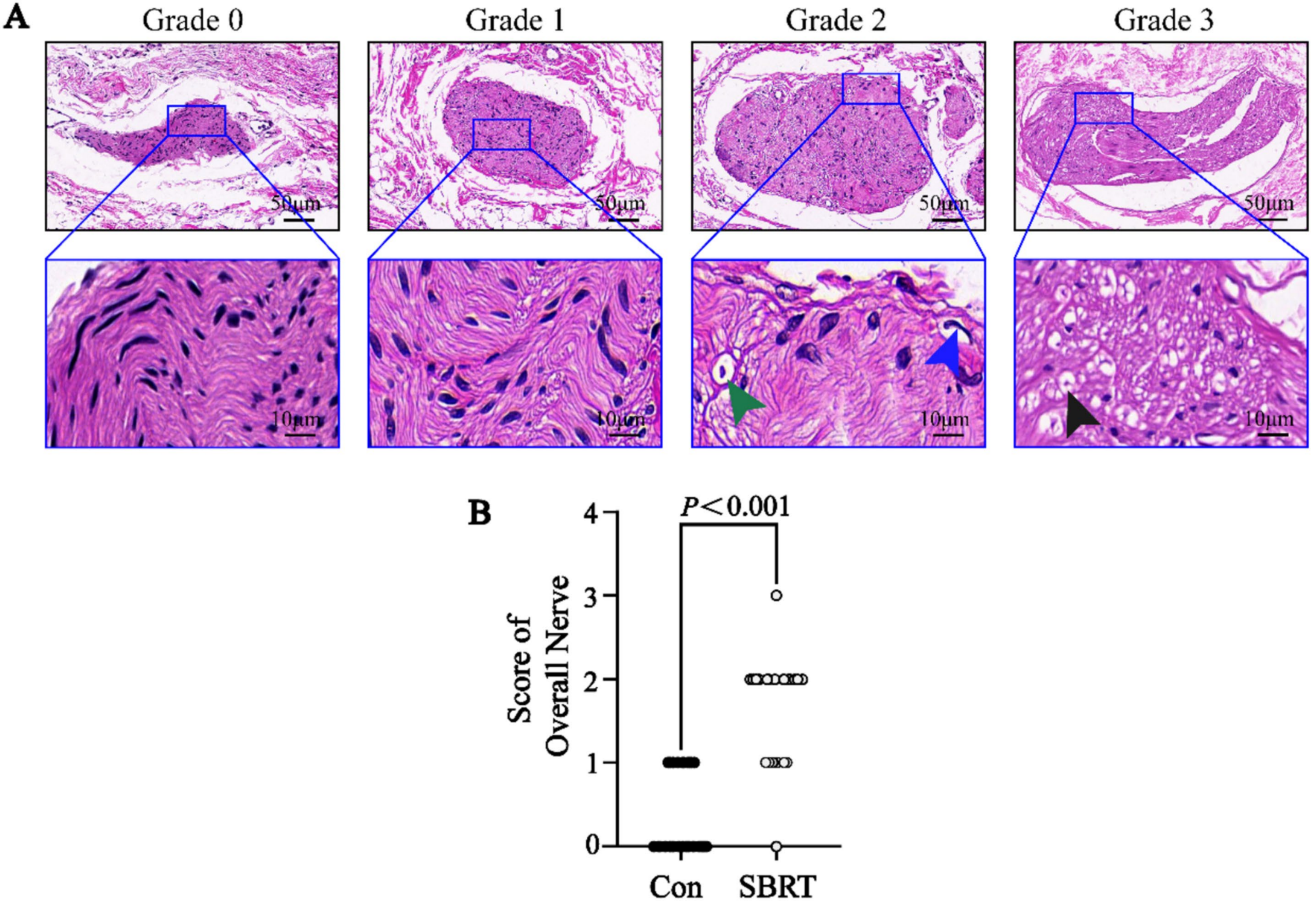
Semiquantitative grading scheme for nerve with hematoxylin and eosin staining. **(A)** Semiquantitative grading scheme for nerve with HE in the SBRT group. (Upper) Representative images of nerves by increasing grade of injury. (Lower) High-power images of injured nerves (dark blue boxed area in upper panel) in each grade. (Lower) Representative images of vacuolization (green arrowhead), pyknotic nuclei (dark blue arrowhead), and digestion chambers (dark arrowhead). **(B)** Semiquantitative nerve injury scores between the control group and SBRT group. HE, hematoxylin and eosin; Con, control group; SBRT, stereotactic body radiotherapy group. Non-normally distributed quantitative variables were compared using the Mann–Whitney *U* test. Comparisons in same group before and after establishing ATEPAH rabbits: *p* < 0.001. *n* = 6 rabbits per group for all analyses.

**Figure 4:**
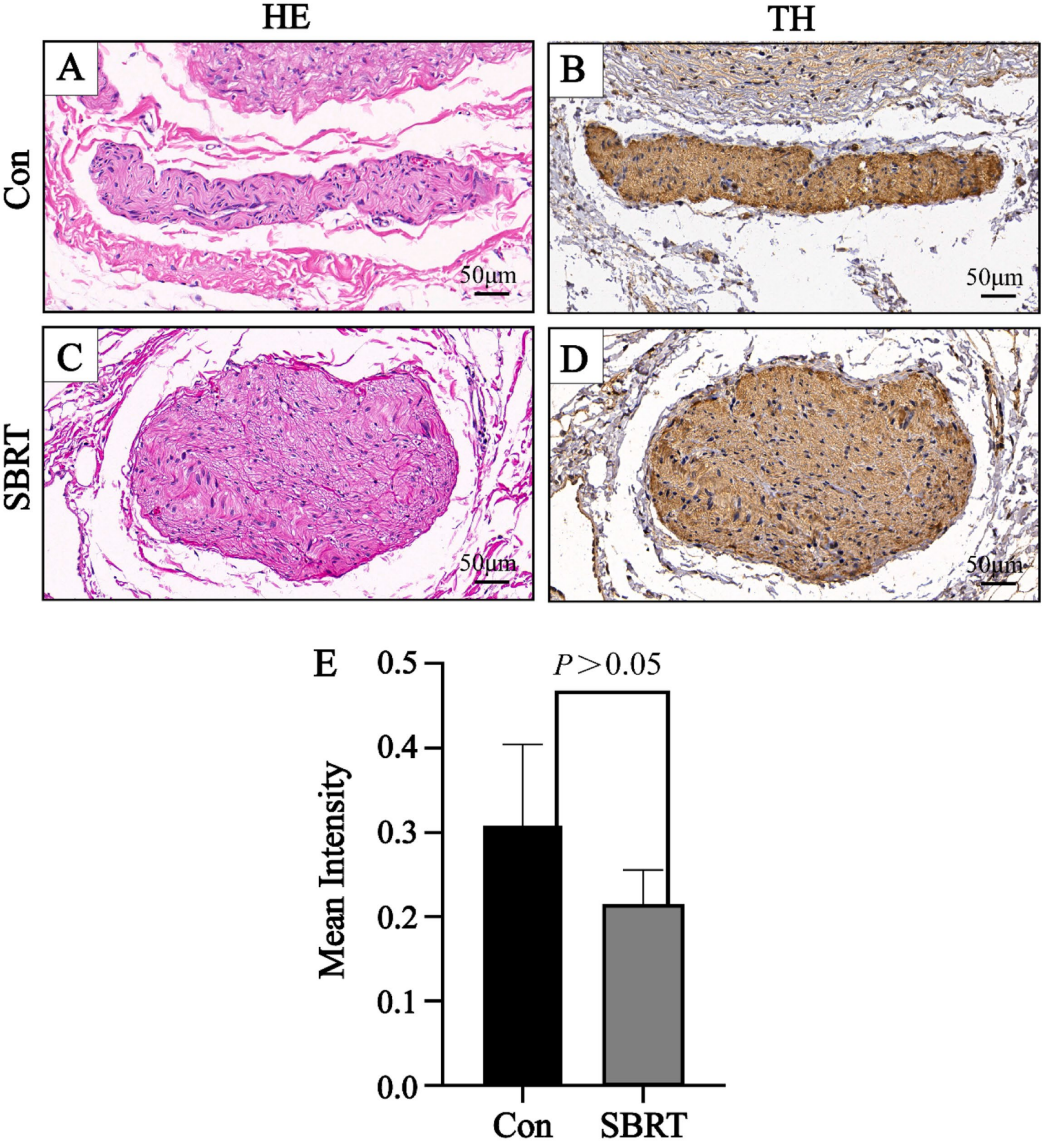
Representative image of nerve with HE and TH. A representative image of nerve with HE **(A)** and a corresponding image stained by TH **(B)** in the control group. A representative image of nerve with HE **(C)** and a corresponding image stained by TH **(D)** in the SBRT group. **(E)** Immunohistochemical staining showed mean intensity of TH of all sympathetic nerves between the control group and SBRT group. HE, hematoxylin and eosin; TH, tyrosine hydroxylase; Con, control group; SBRT, stereotactic body radiotherapy group. Non-normally distributed quantitative variables were compared using the Mann–Whitney *U* test. Comparisons in same group before and after establishing ATEPAH rabbits: *p* > 0.05. *n* = 6 rabbits per group for all analyses.

The study conducted an analysis of the gross anatomical evaluation of lung tissue from established ATEPAH rabbits, as shown in Supplementary Figure 4. The lungs had wedge-shaped infarctions (Supplementary Figure 4A), along with numerous thromboembolic infarcts and thrombi wedged into arterioles at various cross sections of the lung periphery. In addition, normal pulmonary tissues were also observed. However, no thrombi were discovered in the larger pulmonary arteries located in the central section of the lung. Supplementary Figure 4B shows thrombi wedged into an arteriole. The ablation spots were clearly observed on the adventitia of the PA (Supplementary Figures 4C–E). No perforation or rupture of the PA was identified during the studies (Supplementary Figures 4E, F). Histologic analysis was conducted on all pulmonary arteries harvested from the 12 rabbits. Pathological changes were observed in all nerve structures surrounding the PA in the SBRT groups after ablation. A semiquantitative grading of ablation lesions in peri-pulmonary arterial nerves is shown in Figure 3A. Radiation-induced degenerative and necrotic nerve changes included vacuolization, digestion chambers, and nuclear pyknosis. However, perineural fibrosis was not prominently observed. Overall nerve injury score (Figure 3B) in the SBRT group was significantly greater compared with the control group (*p* < 0.001). To assess PA sympathetic innervation, we performed TH staining. Although TH immunoreactivity in the SBRT group was reduced relative to the control group, no statistical difference was found between two groups (*p* > 0.05; Figure 4).

### Safety evaluation

**Figure 5:**
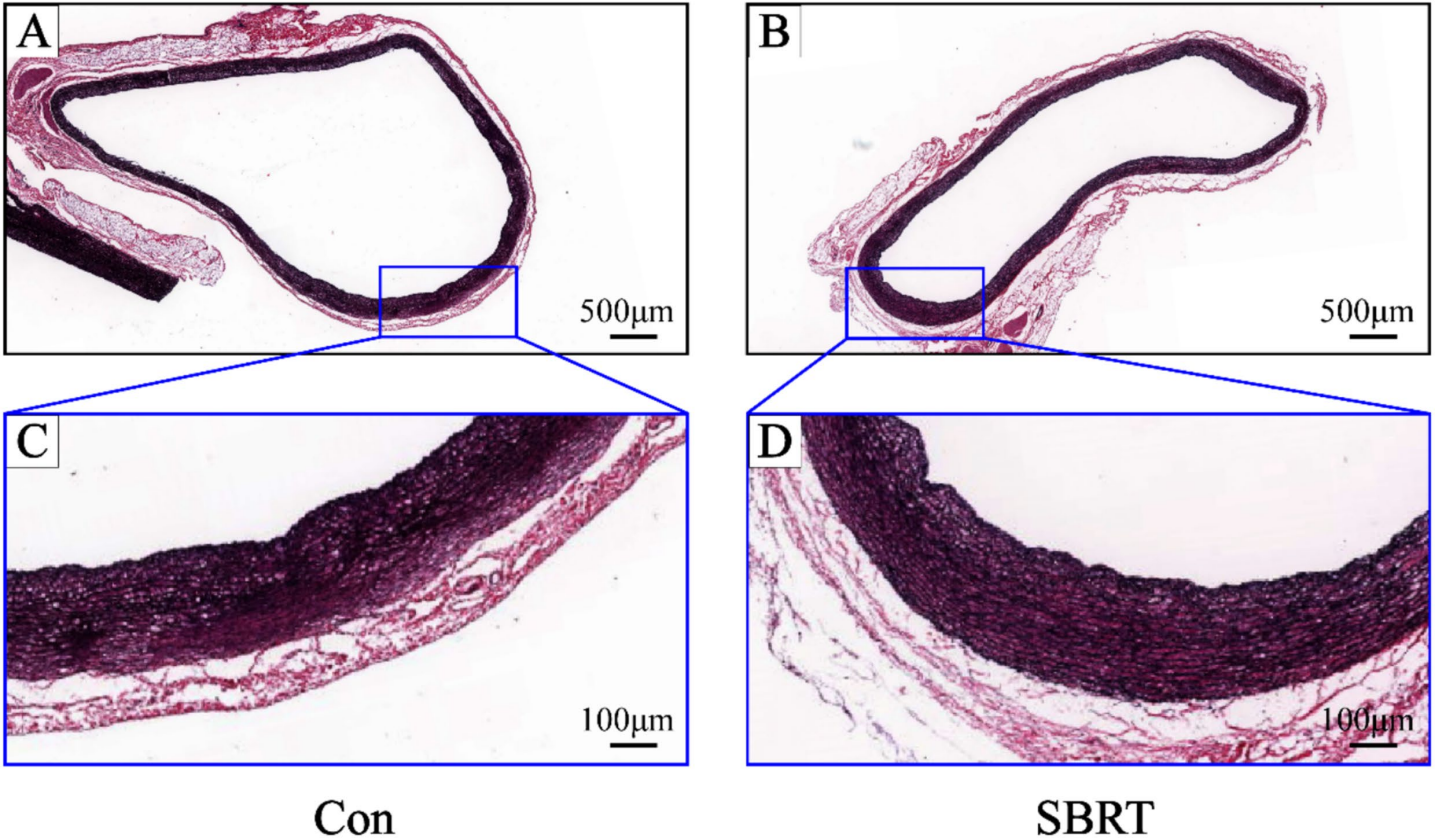
Representative images of pulmonary artery stained by Movat-Russell modified Pentachrome. Representative images of pulmonary artery stained by Movat-Russell modified Pentachrome in the control group **(A)** and SBRT group **(B)**. High-power images of pulmonary artery (dark blue boxed area in upper panel) in the control group **(C)** and SBRT group **(D)**. Pulmonary artery was intact without vascular endothelial cell damage or irregular arrangement of PASMCs, and there was also no evidence of proteoglycan in place of PASMCs. Con, control group; SBRT, stereotactic body radiotherapy group; PASMCs, pulmonary artery smooth muscle cells. *n* = 6 rabbits per group for all analyses.

In the SBRT group, no deaths resulted from radiation exposure. After SBRT treatment, there were no significant changes observed in body weight, mental state, diet, drinking water, defecation, and skin condition of the irradiated area in rabbits. In addition, the general examination revealed no instances of perforation or rupture in PA, heart, and lung. Animals were monitored for 90 days following PADN. A mild pericardial effusion was noted in two of the six animals. No abnormalities were found on activity or weight gain, and cardiorespiratory distress was not detected. Figure 5 shows comparisons of arterial and periarterial tissue injury between two groups. Proteoglycan replacement of pulmonary artery smooth muscle cells (PASMCs) and endothelial loss were not observed in both groups. Pulmonary arterial wall and adjacent tissue injury were barely seen in the SBRT group.

## Discussion

Over the past three decades, significant advancements in the treatment of PAH have been achieved. Particularly significant is the emergence of numerous targeted drugs, which focus on the endothelin, nitric oxide, and prostacyclin pathways. Although these agents may ameliorate clinical symptoms, they are still unable to halt or reverse the progression of PAH, which results in poor long-term prognoses. Several studies have confirmed that PAH was associated with activation of SNS (5–9). PADN is currently under investigation for the treatment of PAH. The current methods for PADN include radiofrequency catheter ablation, ultrasonic catheter ablation, and transthoracic PADN. However, these existing PADN techniques are invasive (4, 10–14). Inspired by the successful application of SBRT in treating hypertension and hypertrophic cardiomyopathy (19, 21, 22), we postulated that it could serve as a promising non-invasive treatment for PADN.

Acute pulmonary arterial hypertension is a clinical syndrome characterized by a rapid increase in pulmonary arterial pressure within a short period of time. Acute pulmonary embolism (APE) caused by venous thromboembolism is the primary cause. Elevated pulmonary arterial pressure results in increased right ventricular afterload and even death (27, 32). One study has shown that rabbit model’s fibrinolytic system was similar to that of human beings (33). Thus, rabbit model was considered as an ideal model for studying APE. After careful consideration, our team ultimately selected rabbits as the experimental animals. Previous experiments, conducted in rats, dogs, porcine, and humans, have demonstrated that SNs surrounding PA primarily distribute in the trunk and bifurcation of the artery (10, 15, 34). Kienecker et al. found that in rabbits, SNs were mainly distributed in the adventitia of PA and partially in the media (35), which provided the basis for our study. Using SBRT, we can accurately and intuitively locate the site that needs to be ablated. We utilized chest contrast-enhanced CT to visualize structure of PA in rabbits. The decision to use this approach was that the naturally faster baseline heart rate of rabbits may influence the resolution ratio and the quality of images based on magnetic resonance imaging (MRI), but chest contrast-enhanced CT with rapid scanning speed and excellent anatomy resolution may overcome the disadvantages of MRI (36–38). Based on the prior research, it has shown that administration of CyberKnife irradiation at a dose of 25 Gy was both efficient and secure in treating arrhythmia (20). One study demonstrated that a single dose irradiation of 15 Gy to planning tumor volume could effectively provide local control and distant control and was comparable with historical controls (18–25 Gy) in terms of efficacy (39). Therefore, in line with the necessary clinical safety measures, the present study employed a single dose irradiation of 15 Gy. The mechanism of SBRT-induced injury is believed to be multifactorial, involving DNA double-strand breaks, cell apoptosis or death, and other factors (22, 40). It takes a longer time for the radiation effect to cause sufficient damage, and previous studies have indicated that the antiarrhythmic effect of radiotherapy requires several days or even months to manifest. In their evaluation of radiotherapy effectiveness, Cuculich et al. established a “window period” of 6 weeks (20). Kennamer et al. found that a single-fraction stereotactic radiosurgery, with greater than or equal to 3 months of follow-up, could effectively provide local control and distant control for patients with metastatic brain tumors (39). A research by Cai et al. suggested that SBRT was safe for renal denervation and could consistently reduce sympathetic nerve activity for up to 6 months (19). Hence, we conducted a 3-month evaluation of our model to assess permanent changes. In this study, the pathophysiological process of pulmonary embolism caused by emboli detachment simulates APE in humans. Furthermore, a rabbit model of ATEPAH was successfully established.

Our study provided preliminary evidence that SBRT improved hemodynamics and attenuated PA dilatation of ATEPAH rabbits in 3 months after PADN. Following APE, right ventricular pressure immediately increased, with both RAD and RVD dilated. Furthermore, the IVS flattened, and PAD widened in both groups. The present findings, which provided evidence regarding the establishment of animal model for acute PAH, were in line with prior research findings (27, 41), but there were no significant improvements observed in RAD, RVD, TAPSE, and PAAT in the SBRT group. In fact, TAPSE showed a more pronounced decrease. The reasons may be as follows: First, the ATEPAH animal model in our study was acute and transient, and the value of PAAT was indeed tend to be improved in the SBRT group than in the control group (25.50 ± 7.64 *VS.* 22.33 ± 5.24; Table 2), although statistical analysis showed that pre- and post-establishing differences between two groups had no statistically significant changes in a short time (*p >* 0.05; Table 2). Second, TAPSE was the common echocardiographic parameter used for quantitative evaluation of RV function, but TAPSE was easily affected by global heart motion or right ventricular after loading conditions, which may be contributed to the poor performance of TAPSE. We need to add extra ultrasonographic index such as RV longitudinal strain to evaluate RV function in our future study. Third, although the values of post-operation RA and post-operation RV in the SBRT group seemed to be higher than in the control group, statistical analysis show that *p-*value of pre- and post-establishing differences between the control and SBRT groups were 0.2083 and 0.0667, respectively (Table 2), which represent the differences were no statistically significant. The elevation of PASP and mPAP after embolization was lower in the SBRT group than in the control group. These results demonstrated that experimental method of SBRT-PADN utilized in this study effectively decreased the elevation of PASP and mPAP caused by APE. This finding aligned with previous research on PADN in PAH (15, 17, 34, 42), highlighting that SBRT-PADN improved hemodynamics in an acute PAH model by declining PASP and mPAP. Moreover, the SBRT group exhibited a lower increase in PA dilatation after embolization compared with the control group. This finding indicated that SBRT-PADN had the potential to mitigate PA dilatation in ATEPAH rabbits.

During the gross specimen examination, pale pink or red ablative foci were identified in PA of the SBRT group, while no ablation lesions were observed in surrounding lung tissue or heart. To further confirm the validity of our study, we performed histological evaluations. In this study, the SBRT group was able to observe ablation injury to nerve tissue of PA. This injury was categorized into 0 to 3 grades using a semi-quantitative scoring system. Grade 0 indicated no damage, while grade 1 indicated minimal injury with little to no vacuolization, pyknotic nuclei, suggesting that the nerve was mostly intact and may still function. For grade 2, there were more obvious changes in neurons, such as vacuolization and pyknotic nuclei. Grade 3 indicated a moderate level of injury with significant changes in the endoneuronal damage, including frequent pyknotic nuclei, digestion chambers, and vacuolization (28). These findings suggested that SBRT-PADN might induce degenerative and necrosis changes, such as pyknotic nuclei, digestion chambers, and vacuolization, in the perineuronal and/or intra-neuronal parts of the PA nerve. In addition, we detected the rate-limiting enzyme TH in PA, which was involved in catecholamine synthesis. This allowed us to indirectly evaluate neurotransmitter synthesis and obtain an indicator of sympathetic nerve activity in PA (43). In our study, we observed a weakening TH staining of SNs around PA after PADN.

In recent years, PADN has emerged as a novel method for treating PAH, with a majority of the current procedures being invasive in nature. To investigate an alternative approach, our team explored high-intensity focused ultrasound (HIFU) for PADN through ablation. The study determined that HIFU could improve hemodynamics of rabbits with ATEPAH, which corroborates earlier research findings (18). This technique could be deemed successful in achieving denervation of the PA. However, in the ablation group, ablation foci were identified in the lung tissue of four rabbits, indicating the necessity for further improvement in accuracy (18). To address this issue, our study employed SBRT for performing PADN, and aligning with the outcomes of HIFU-PADN, hemodynamics of rabbits with ATEPAH were improved post-SBRT-PADN.

Safety is a paramount concern for novel PADN; in SBRT-PADN, the greatest challenge is to mitigate the potential risk of damaging adjacent tissues. In our study, we made concerted efforts to minimize any unwanted radiation-induced injuries to surrounding normal tissues. The target volume was defined using chest contrast-enhanced CT. PA from the ostium to lung hilum could be contoured and visualized on CT images prior to treatment. No perforation or rupture of PA, heart, and lungs has been observed; meanwhile, no skin or other tissue ablation damage was identified in our study. Pulmonary arterial wall injuries were barely seen in the SBRT group. The preliminary results presented above indicated that SBRT-PADN was a precise and safety treatment option.

## Study limitations

As an exploratory preclinical study, this research gave priority to short-term safety assessments because of the intrinsic difficulties in rabbit rearing and experimental limitations. The current study only reported that SBRT-PADN treatment abolished the acute experimental PAH. The effect of PADN treatment in chronic pulmonary hypertension or other types unsettled. In future studies, we intend to expand the sample size and conduct further preclinical trials to determine the appropriate dosage and optimum follow-up period before human clinical trials can be conducted. However, the safety of SBRT-PADN treatment was approved as no experimental rabbits died after PADN treatment.

## Conclusion

SBRT could damage SNs around PA locally in preclinical rabbit model, demonstrating the feasibility and effectiveness of non-invasive stereotactic PA nerve ablation. The optimal radiation dosage of 15 Gy exhibited the safest short-term profile while maintaining efficacy in this study. This technique offered a potential alternative to PADN. However, due to the early stage of SBRT evaluation for PADN, further investigation and preclinical experience with longer follow-up are necessary before implementation in clinical practice. Additional research is needed to investigate the potential effects of SBRT on the inducibility of PAH in chronic experiments.

## Data Availability

The original contributions presented in the study are included in the article/Supplementary material, further inquiries can be directed to the corresponding author.
