# Supplementary material for: Stereotactic body radiotherapy: a new non-invasive way to conduct pulmonary artery denervation
Source: Front Med (Lausanne). 2025 Jun 25;12:1607638. doi: 10.3389/fmed.2025.1607638 (PMC12238088; doi:10.3389/fmed.2025.1607638)
Supplement: Supplementary file 1 [file Data_Sheet_1.docx]

**Supplemental Material**

**Supplemental Table 1 Treatment Characteristics**

| **Case**  **No.** | **Dose,**  **Gy** | **Follow-up,**  **month(s)** | **Treatment**  **Time, min** | **PTV**  **(Pulmonary Artery)** | | |  | **Maximum/Mean Doses to Major OARs, Gy** | |
| --- | --- | --- | --- | --- | --- | --- | --- | --- | --- |
|  |  |  |  | **Volume,**  **Ml** | **Maximum**  **Dose, Gy** | **Minimum**  **Dose, Gy** |  | **Spinal Cord** | **Lung** |
| 1 | 15 | 3 | 5.6 | 1.9 | 16.2 | 13.5 |  | 8.8/5.1 | 15.2/1.3 |
| 2 | 15 | 3 | 7.4 | 1.9 | 16.1 | 14.3 |  | 9.0/7.4 | 15.7/1.1 |
| 3 | 15 | 3 | 6.6 | 2.2 | 16.2 | 13.7 |  | 9.5/3.4 | 15.7/1.5 |
| 4 | 15 | 3 | 6.8 | 2.4 | 16.0 | 13.8 |  | 9.5/5.1 | 15.8/2.0 |
| 5 | 15 | 3 | 8.3 | 2.4 | 16.0 | 13.8 |  | 8.2/5.5 | 15.8/1.7 |
| 6 | 15 | 3 | 7.3 | 3.3 | 15.9 | 13.8 |  | 8.1/7.3 | 15.8/1.6 |

OARs = organs at risk; PTV = planning target volume; Gy = Gray.

**
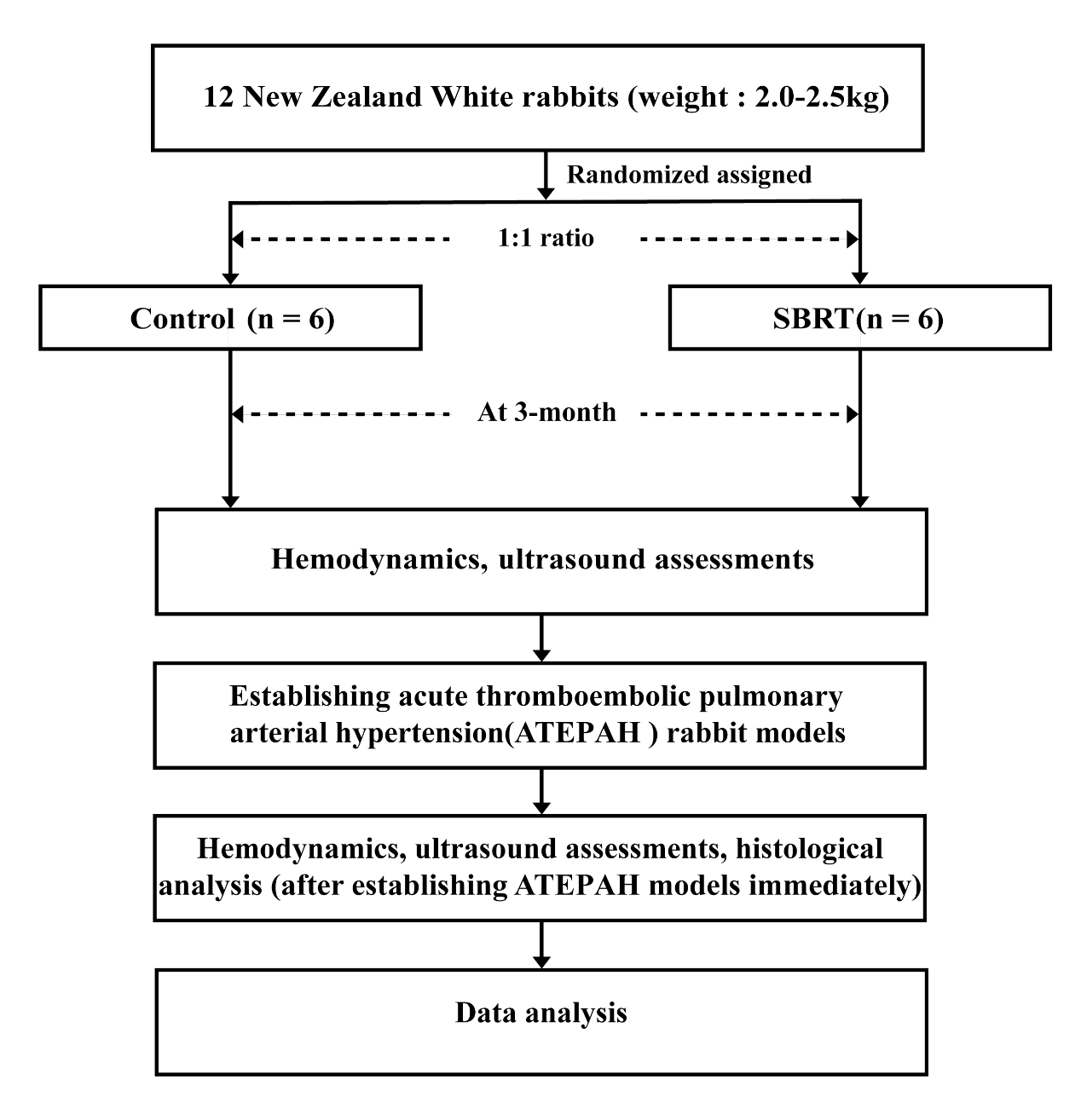
**

**Supplemental Figure 1 Study flowchart.**

12 New Zealand rabbits were randomly divided into control group and SBRT group. Rabbits in the SBRT group underwent SBRT and were bred for a minimum of 3 months. Hemodynamics and ultrasonography parameters were measured using RHC and echocardiography before and after establishing ATEPAH models in both groups. Then MPA and bifurcation of PA were harvested immediately after completing RHC for subsequent histological analysis and immunohistochemistry of tyrosine hydroxylase. SBRT = stereotactic body radiotherapy; RHC = right heart catheterization; ATEPAH = acute thromboembolic pulmonary arterial hypertension; MPA = main pulmonary artery; PA = pulmonary artery. n = 6 rabbits per group for all analyses.

**
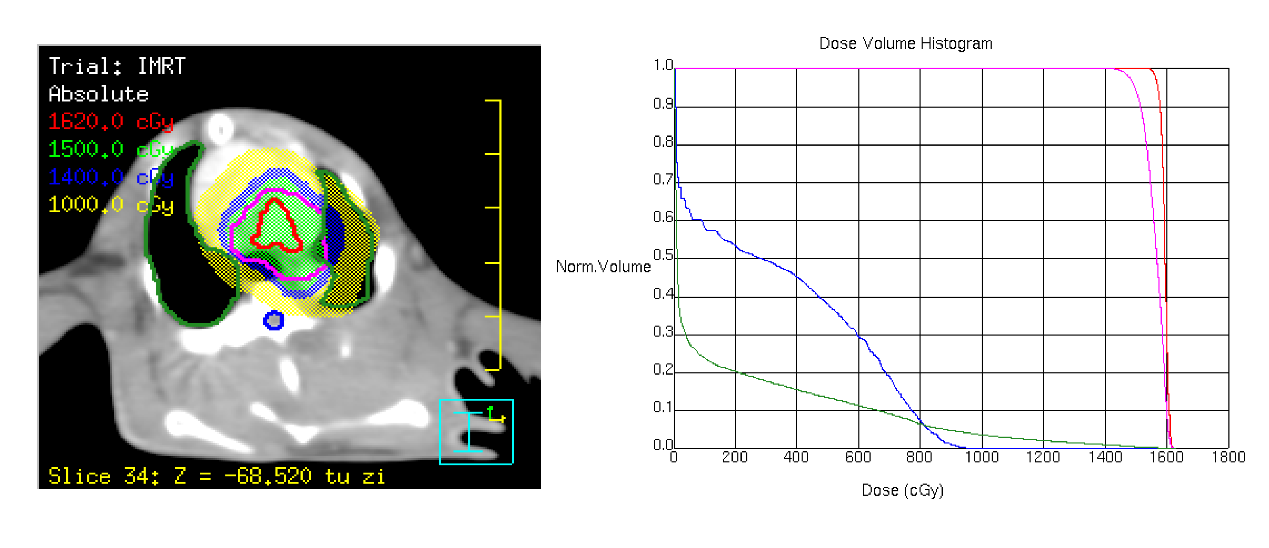
**

**1cGy=0.01Gy**

**Supplemental Figure 2 Screenshot of stereotactic body radiotherapy planning system.**

Views of contouring outcome (**left panel**) and dose volume histogram (**right panel**) demonstrates the designated dose concentrates on the planning target areas around the pulmonary arteries with a rapid dose falloff to adjacent critical structures, including spinal cord (**dark blue**) and lungs (**green**). Gy= Gray.

**
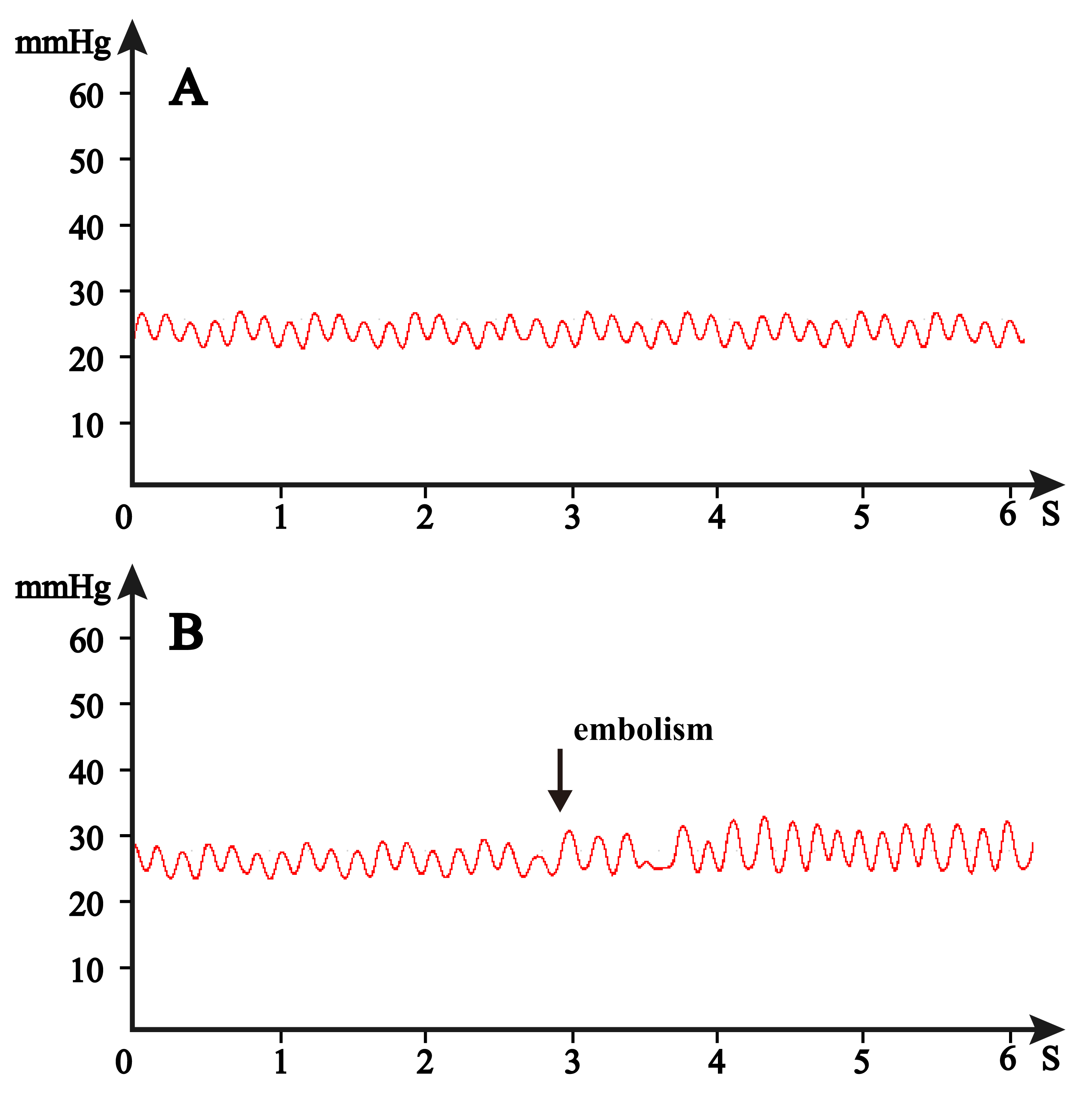
**

**Supplemental Figure 3 Comparison of pulmonary artery systolic pressure pre- and post-embolization.**

(A) Normal pulmonary artery systolic pressure waveform. (B) Increased pulmonary artery aystolic blood pressure after embolization.

**
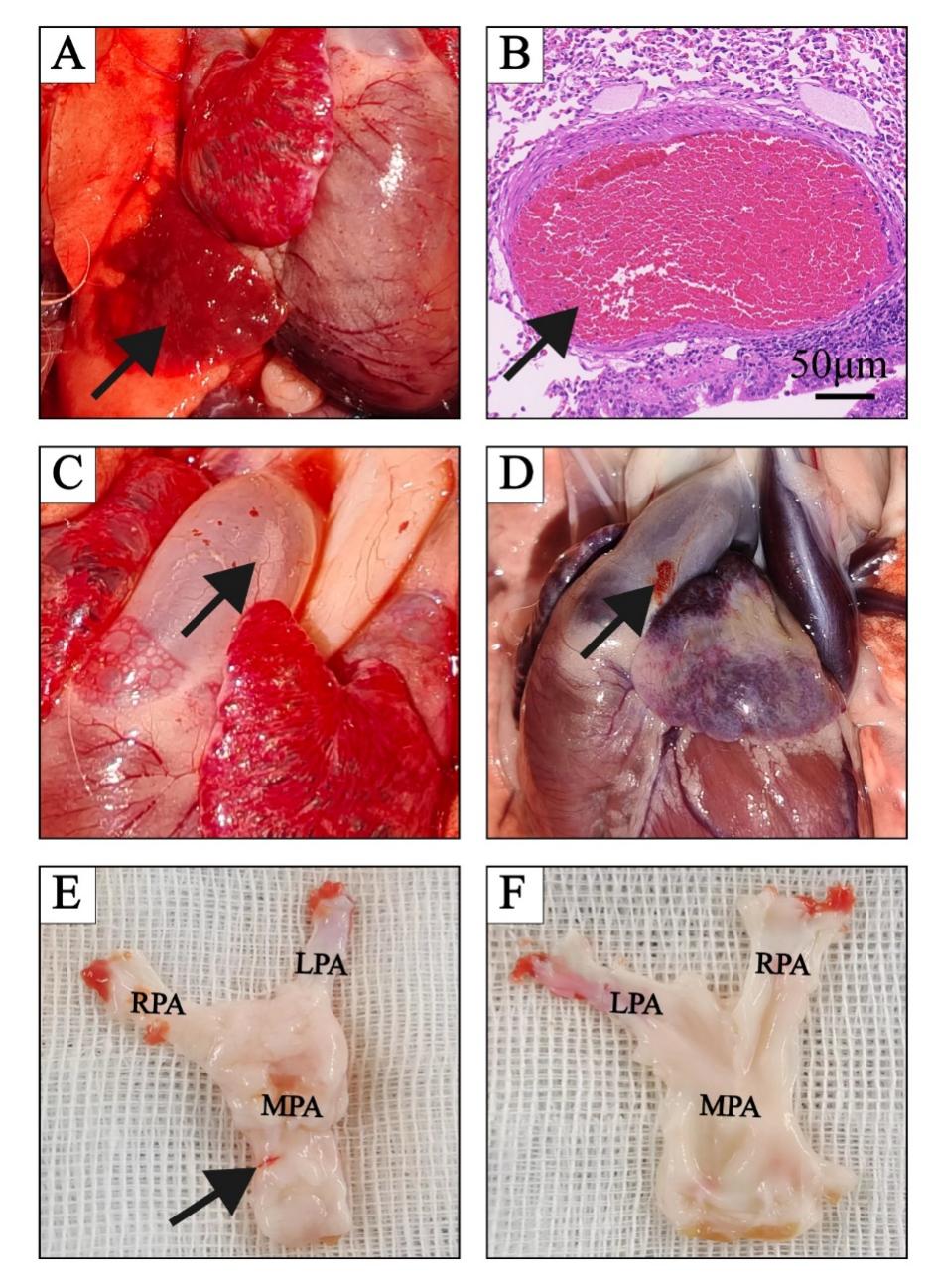
**

**Supplemental Figure 4** **The gross specimen of lung tissue after establishing ATEPAH rabbits.** The gross specimen of lung tissue after establishing ATEPAH rabbits. (**A**) An obvious segmental infarct in the lung tissue. (**B**) HE histological section of lung tissue showed thrombi wedged into an arteriole. (**C**) Arrows indicate flaky pale-red ablation foci on the MPA adventitia. (**D**) Arrows indicate flaky red ablation foci on the MPA adventitia. (**E**) Arrows indicate linear red ablation foci on the right side of MPA adventitia. (**F**) No lesions are visible on the intima of the PA trunk, the left and right PA branches. ATEPAH = acute thromboembolic pulmonary arterial hypertension; HE = hematoxylin and eosin; MPA = main pulmonary artery; PA = pulmonary artery; LPA = left pulmonary artery; RPA = right pulmonary artery.
